# The Chemical Components of *Sesbania grandiflora* Root and Their Antituberculosis Activity

**DOI:** 10.3390/ph5080882

**Published:** 2012-08-23

**Authors:** Noviany Hasan, Hasnah Osman, Suriyati Mohamad, Keng Chong Wong, Khalijah Awang, Anis Safirah Mohd Zahariluddin

**Affiliations:** 1 School of Chemical Sciences, University of Science Malaysia, Minden 11800, Penang, Malaysia; Email: novyany73@gmail.com (N.H.); kcwong@usm.my (W.K.C.); 2 Department of Chemistry, University of Lampung, Bandar Lampung 35145, Lampung, Indonesia; 3 School of Biological Sciences, University of Science Malaysia, Minden 11800, Penang, Malaysia; Email: suri@usm.my (S.M.); anissafirah@yahoo.com (A.S.M.Z.); 4 Department of Chemistry, Faculty of Science, University of Malaya, Kuala Lumpur 50603, Malaysia; Email: khalijah@um.edu.my (K.A)

**Keywords:** isovestitol, medicarpin, sativan, betulinic acid, *Sesbania grandiflora*, antituberculosis activity, *Mycobacterium tuberculosis*

## Abstract

Three isoflavanoids, isovestitol (**1**), medicarpin (**2**), and sativan (**3**), along with another known compound, betulinic acid (**4**), were isolated from the root of *Sesbania grandiflora*. The structures of the isolated compounds were characterised by means of spectroscopic techniques (UV, IR, MS, ^1^H- and ^13^C-NMR, DEPT, COSY, HMQC, HMBC, and MS analysis). All the tested compounds **1**–**4** exhibited antituberculosis activity against *Mycobacterium tuberculosis* H37Rv, with MIC values of 50 µg/mL for compounds **1–3**, and 100 µg/mL for compound **4**, whereas, the methanol extract exhibited antituberculosis activity of 625 µg/mL. This is the first report on the occurrence of isoflavonoids in this plant and their antituberculosis activity.

## 1. Introduction

*Sesbania grandiflora* (L.) Pers. is a small, erect, fast-growing, and sparsely branched tree belonging to the Leguminosae family. This plant is native to tropical Asia and is widespread in Malaysia, Indonesia, Philippines, and India. The Malay names of this plant are turi and geti. All parts of *S. grandiflora* have been used empirically as a traditional remedy in folk medicine to treat various diseases such as catarrh, dysentery, fevers, headaches, smallpox, sore throat, and stomatitis [1,2].

Previous phytopharmacological study on the leaves, flowers, and aerial parts of this plant had isolated sterols, saponins, and tannins [3]. These chemical constituents are well known for their potential health benefits and have been reported to possess valuable biological activities such as antibacterial and antifungal [4], antioxidant [5,6,7], antiurolithiatic [7], anticonvulsant and anxiolytic [8], and hepatoprotective properties [9]. In a more recent study, it was found that the supplementation of *S. grandiflora* leaves could also afford a significant hypolipidemic effect against Triton-induced hyperlipidemia in rats [10]. Even though *S. grandiflora* was extensively studied by other researchers for its phytopharmacological potential, especially the leaves, flowers, and aerial parts of the plant, no phytochemical and pharmacological studies have been performed on the root of *S. grandiflora*.

We report herein the phytochemical investigation of the methanol and acetone extracts of *S. grandiflora* roots, which led to the isolation and identification of three isoflavanoids: isovestitol (**1**), medicarpin (**2**), and sativan (**3**), together with the known betulinic acid (**4**). All isolated compounds were evaluated for their inhibitory activity towards the growth of *M. tuberculosis* H37Rv. This is the first report of the four compounds isolated from the root of *S. grandiflora* and their antituberculosis properties.

## 2. Results and Discussion

### 2.1. Structure Elucidation

The MeOH-soluble fraction and EtOAc-soluble fraction of the MeOH extract of *S. grandiflora* root afforded four compounds **1**–**4**, after repeated column chromatography purifications. Compound **1** was isolated as an amorphous powder, [α]_D_^20^: −66.6 (MeOH). The molecular formula of **1** was determined as C_16_H_16_O_4_ ([M+H]^+^*m/z* 273.1) from the FAB mass spectrum. This compound was found to be an isoflavan on the basis of its characteristic spectral data: λ_max_ 227 and 284 nm in the UV spectrum and a set of aliphatic proton signals (δ 2.81, 2.98, 3.49, 3.99, and 4.25) in the ^1^H-NMR spectrum, which in addition displayed three aromatic protons in an AMX system (δ 6.43, 6.51, and 7.06) and three aromatic protons in an ABM system (δ 6.29, 6.38, and 6.90). The ^13^C-NMR spectrum exhibited signals for 17 carbons which were distributed between one methoxyl, two methylenes, seven methines, and six quaternary carbons. The molecular structure of **1** was confirmed by a DEPT experiment. Further assignment was done by Heteronuclear Multiple Quantum Coherence (HMQC) and Heteronuclear Multiple Bond Correlation (HMBC) spectra. The placement of one methoxyl group and two hydroxyl groups at the C-2', C-4', and C-7 positions, respectively, were confirmed from the HMBC experiment, which revealed a correlation between the methoxyl group with a carbon at C-2' (δ 159.64), and a correlation between the hydroxyl group at C-4' (δ 155.64) and carbon at C-3' (δ 102.08) and C-5' (δ 102.08). The position of the other hydroxyl group was assigned at C-7. The HMBC spectrum exhibited a correlation between the hydroxyl group at C-7 (δ 157.03) and carbon at C-6 (δ 108.28), and C-8 (δ 103.99). On the basis of the spectroscopic evidence, compound **1** was characterised as 7,4'-dihydroxy-2'-methoxyisoflavan or isovestitol [11,12].

Compound **2** was obtained as an amorphous powder and its molecular formula was assigned as C_16_H_14_O_4_ ([M-H]^+^*m/z* 269.0816) from the HRESI mass spectrum. The characteristic spectral data; λ_max_ 229 and 286 nm in the UV spectrum and a pair of four aliphatic protons (δ 3.61; 3.61; 4.28; and 5.52) in the ^1^H-NMR spectrum revealed that compound **2** has a pterocarpan skeleton. The ^1^H-NMR spectrum (Table 1) of compound **2** revealed two sets of the AMX type aromatic protons (δ 6.37, 6.57, and 7.33; and 6.39, 6.46, and 7.24), one methoxyl group (δ 3.76, 3H), and one hydroxyl group (δ 8.66). The location of the methoxyl group at C-9 and the hydroxyl group at C-3 position were assessed by a HMBC experiment. The structure of compound **2** was deduced from detailed analysis of ^1^H-and ^13^C-NMR data aided by 2D-NMR experiments (COSY, HMQC, HMBC, and NOESY) and identified as 3-hydroxy-9-methoxypterocarpan or medicarpin [12].

**Table 1:** ^1^H- and ^13^C-NMR data (aceton-*d_6_*, 400; 300 MHz resp.) of compound **1**–**3**. δ in ppm, *J* in Hz.

Atom	1		2		3	
	δ (H)	δ (C)	δ (H)	δ (C)	δ (H)	δ (C)
5	6.90, *d* (8.2)	130.49	-		6.90, *d* (8.2)	130.47
6	6.38, *dd *(8.2 & 2.4)	108.28	H_α_, 4.28, *br d * (6)	66.6	6.38, *dd *(8.2 & 2.5)	108.00
	-	-	H_β_, 3.61, *d* (6)		-	
7	-	157.03	7.24, *d* (8.2)	125.3	-	157.08
8	6.29, *d *(2.4)	103.99	6.46, *dd* (8.2 & 2.3)	106.4	6.29, *d *(2.5)	103.21
9	-	156.23	-	161.6	-	155.59
10	-	113.83	6.39, *d* (2.3)	96.8	-	113.00
1'	-	120.49	-	-	-	122.11
2'	-	159.64	-	-	-	158.71
3'	6.51, *d* (2.5)	102.08	-	-	6.59, *d* (2.5)	98.88
4'	-	155.64	-	-	-	160.33
5'	6.43, *dd* (8.5 & 2.5)	105.24	-	-	6.50, *dd* (8.4 & 2.5)	105.04
6'	7.06, *d* (8.5)	128.24	-	-	7.10, *d* (8.4)	127.92
1	-	-	7.33, *d* (8.4)	132.6	-	-
2	H_α_, 3.99, *t* (10)	70.01	6.57, *dd *(8.2 & 2.4)	110.0	H_α_, 3.95, *t *(10)	70.07
	H_β_, 4.25, *brd* (10; 3; & 2)	-	-	-	H_β_, 4.20, *ddd* (10; 3; & 2)	-
3	3.49, *m* (8; 5; & 3)	33.69	-	159.2	3.47, *m*	31.97
4	H_α_, 2.81, *dd* (10; 5 & 2)	32.19	6.37, *d* (2.4)	103.5	H_α_, 2.78, *br d* (7; 5 & 2)	30.68
	H_β_, 2.98, *dd* (16, 5)	-	-	-	H_β_, 2.81, *br d* (7; 5 & 2)	-
4a	-	-	-	157.3	-	-
6a	-	-	3.61, *d* (6)	39.9	-	-
6b	-	-	-	119.9	-	-
10a	-	-	-	161.3	-	-
11a	-	-	5.52, *br d *(6)	78.9	-	-
11b	-	-	*-*	112.4	-	-
3-OH	-	-	8.66, *br s*	-		
2'-OCH_3_	3.73, *s*, 3H	54.89	-	-	3.80, *s*, 3H	55.07
4'-OH	8.14, *br s*	-	-	-	-	-
4'-OCH_3_	*-*	-	-	-	3.86, *s*, 3H	55.36
7-OH	8.59, *br s*	-	-	-	8.15, *br s*	-
9-OCH	*-*	-	3.76, *s* , 3H	55.2	*-*	-

Compound **3** was obtained as an amorphous powder and its molecular formula was analysed as C_17_H_18_O_4_ ([M-H]^+^*m/z* 285.1119) from the HRESI mass spectrum. The spectral (IR, ^1^H-NMR and ^13^C-NMR) data of compound **3** revealed that its structure was similar to that of the isolated compound **1** mentioned earlier in this paper. The hydroxyl group at the C-4' position of **1** is replaced by a methoxyl group in **3**. It was also clearly observed that the ^13^C-NMR spectrum of **3** (Table 1) revealed two methoxyl carbons at δ 3.76 and 3.86. Thus, the structure of compound **3** was confirmed as 7-hydroxy-2',4'-dimethoxyisoflavan or sativan [13]. This structure was further confirmed from ^1^H-^1^H COSY and HMBC experiments.

Another known isolated compound, 3β-hydroxy-lup-20(29)-en-(28)-oic acid or betulinic acid (**4**) was readily identified based on its spectroscopy data (1D-and 2D-NMR) and also by comparing its physico-chemical, spectroscopic, and mass spectrometric data with literature values [14].All isolated compounds are known compounds, therefore, elucidation of their structures was confirmed by comparison of their spectral data with those reported earlier.

### 2.2. Biological Assay

The crude extract of *S. grandiflora* root and the isolated compounds were assessed *in vitro* against *M. tuberculosis* H37Rv. The antituberculosis activity data are displayed in Table 2. A first-line antituberculosis drug, isoniazid, was used as a positive control. The methanol extract exhibited a moderate activity with MIC value of 625 µg/mL. Among the isolated chemical constituents, compounds **1**, **2** and **3** showed similar antituberculosis activity, with MIC values of 50 µg/mL, while compound **4** exhibited a weaker activity, with a MIC value of 100 µg/mL. This is the first report on the antituberculosis activity of the tested compounds.

**Table 2:** Antituberculosis activity of extract and compounds **1**–**4** against *M. tuberculosis* H37Rv.

Test Sample	MIC ± SD
MeOH extract	625 ± 0.0 µg/mL
Isovestitol (**1**)	50 ± 0.0 µg/mL
Medicarpin (**2**)	50 ± 0.0 µg/mL
Sativan (**3**)	50 ± 0.0 µg/mL
Betulinic Acid (**4**)	100 ± 0.0 µg/mL
Isoniazid	0.078 ± 0.0 µg/mL

## 3. Experimental

### 3.1. General

TLC was performed on pre-coated Merck 60 GF_254_ silica gel plates (absorbent thickness, 0.25 mm) and sprayed with Ce (IV) sulphate solution for visualization of spots. Preparative plates TLC was performed on 20 cm × 20 cm glass plates coated with 0.5 mm Kieselgel F_254_ (Merck), and were air-dried and used without prior activation. Column chromatography was performed on silica gel (Merck Kieselgel 60, 70-230 mesh ASTM). Melting points were recorded on a Stuart Scientific SMP1 apparatus. Optical rotations were determined using a JASCO DIP–370 digital polarimeter with a 0.5 cm microcell. NMR spectra were recorded in acetone-d_6_, with TMS as internal standard, using either a Bruker Avance 300 or a Bruker Avance 400 spectrometer, HRESIMS spectra were using a Micro TOF-Q mass spectrometer in positive-ion mode. IR(KBr) spectra were recorded using a Perkin-Elmer system 2000 FT-IR spectrometer. UV spectra were recorded using a Perkin-Elmer Lambda 25 spectrometer. Blank discs and antibiotic discs were purchased from Oxoid, Middlebrook 7H9 broth from Difco, and Tetrazolium-Tween 80 mixture from Sigma.

### 3.2. Plant Material

The roots of *Sesbania grandiflora* were collected in September 2008 in the village of Sidosari, South Lampung, Indonesia. The identity of the plant specimen was authenticated by Harry Wiriadinata and a voucher specimen (No. N-III) was deposited at the Bogoriense Herbarium, Bogor, Indonesia.

### 3.3. Extraction and Isolation

**Figure 1:**
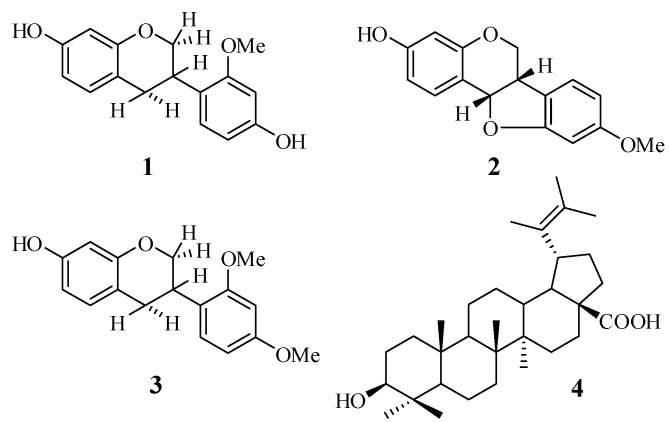
Chemical structures of the isolated compounds **1–4**.

**Figure 2:**
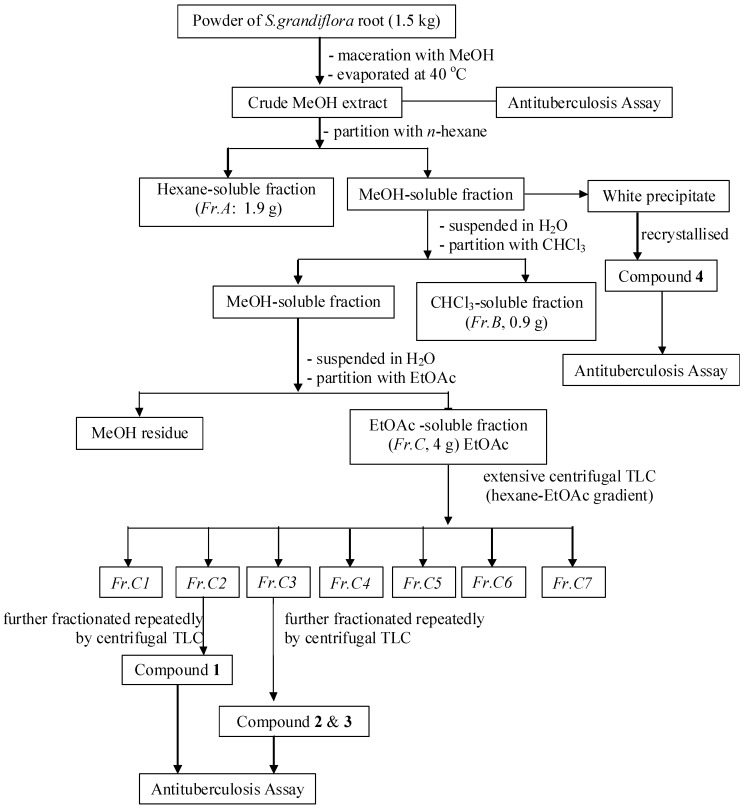
Flow chart of extraction and isolation.

Air***-***dried and powdered roots (1.5 kg) of *S. grandiflora* were extracted with 90% aqueous MeOH (3 × 5 L) at room temperature for a period of seven days. The extract was concentrated to a volume of 100 mL under reduced pressure using a rotatory evaporator at a bath temperature of 40 °C, and then partitioned with hexane to afford a hexane-soluble fraction (*Fr. A*, 1.9 g) and a MeOH-soluble fraction. The MeOH-soluble fraction was suspended in H_2_O and partitioned sequentially with CHCl_3_, and EtOAc, yielding a CHCl_3_-soluble fraction (*Fr. B*, 0.9 g) and an EtOAc-soluble fraction (*Fr. C*, 4 g). *Fr. C* was separated by extensive centrifugal TLC (hexane-EtOAc gradient) to afford 7 major fractions, *Fr. C1*–*C7*. *Fr*. *C2* (290 mg) was further fractionated by centrifugal TLC (CHCl_3_-MeOH 95:5 v/v) to afford 8 sub-fractions, *Fr*. *C2.1–Fr. C2.8.* *Fr. C2.6* (180 mg) was fractionated by column chromatography (hexane-EtOAc 95:5 v/v) to give 10 sub-fractions: *Fr.C2.6.1–Fr*. *C2.6.10*. *Fr. C2.6.7* (40 mg) was purified by silica gel preparative TLC (hexane-CHCl_3_-MeOH 5:99:1 v/v/v) to afford **1** (20 mg, *R_f_* 0.25). *Fr*. *C3 *(250 g) was separated by centrifugal TLC (CHCl_3_-MeOH gradient) to afford 7 sub-fractions, *Fr.C3.1–Fr. C3.7. Fr.C3.4* (95 mg) was further purified by preparative silica gel TLC (toluene-EtOAc 99:1 v/v) to yield **2** (18.5 mg, *R_f_* 0.45) and **3** (7 mg, *R_f_* 0.47). Compound **4** (23.7 mg) was obtained by recrystallization from the MeOH-soluble fraction. The fractionation procedure is summarized in Figure 1. The isolated compounds **1**–**4** were readily identified based on their spectroscopic data (1D- and 2D-NMR) and also by comparing their physico-chemical, spectroscopic, and mass spectrometric data with those reported in the literature. All the isolated compounds are known compounds, and were confirmed as isovestitol (**1**), medicarpin (**2**), sativan (**3**) and betulinic acid (**4**), the structures of which are shown in Figure 2.

### 3.4. Antituberculosis Activity Assay

Assay was performed by the Tetrazolium Microplate Assay method as described previously [15], with minor modifications. A susceptible virulent strain of *M. tuberculosis* H37Rv ATCC 27294 was used in this study. The test concentrations of purified compounds and the extracts ranged from 200 to 0.391 μg/mL and 2,500 to 4.883 μg/mL, respectively. Each sample was tested in triplicates twice. The plates were sealed with Parafilm and incubated at 37 °C in 8% CO_2_ for 5 days. On day 5, Tetrazolium-Tween 80 mixture {50 μL, 1.5 mL of tetrazolium bromide [3-(4,5-dimethylthiazol-2-yl)-2,5-diphenyl-tetrazolium bromide] at a dilution of 1 mg/mL in absolute ethanol and 1.5 mL of sterile 10% Tween 80} was added to a control well and then reincubated at 37 °C for 24 h. Tetrazolium-Tween 80 mixture was added to all wells and the colour was recorded at 24 h. A colour change from yellow to purple indicated growth and the minimum inhibitory concentration (MIC) was interpreted as the lowest tested compound and extract concentration which prevented the colour change from yellow to purple.

## 4. Conclusions

Extract and isolated compounds from the root of *S. grandiflora* possessed activity against *M. tuberculosis* H37Rv. The methanol extract exhibited a moderate activity, while the isoflavanoid compounds showed promising antitubercular activity. The present study is the first report of the antimicrobial activity of the chemical components and extract from the root of *S. grandiflora*. The results of this study can be used as additional data for future development and utilization of *S. grandiflora.*
